# To predict the left ventricular endocardial scar tissue pattern using Radon descriptor-based machine learning

**DOI:** 10.1186/s13104-023-06466-0

**Published:** 2023-08-24

**Authors:** Yashbir Singh, Shadi Atalla, Wathiq Mansoor, Rahul Paul, Deepa Deepa

**Affiliations:** 1 Biomedical Engineering, Chung Yuan Christian University, Zhongli, Taiwan; 2 Department of Radiology, Mayo clinic, Rochester, MN USA; 3 Engineering & Information Technology, University of Dubai, Dubai, United Arab Emirates; 4 Department of Radiation Oncology, Massachusetts General Hospital, Boston, USA

**Keywords:** Atrial fibrillation, Radon descriptors, Left ventricle, Machine learning, Morphological operation

## Abstract

**Objective:**

Scar tissue is an identified cause for the development of malignant ventricular arrhythmias in patients of myocardial infarction, which ultimately leads to cardiac death, a fatal outcome. We aim to evaluate the left ventricular endocardial Scar tissue pattern using Radon descriptor-based machine learning. We performed automated Left ventricle (LV) segmentation to find the LV endocardial wall, performed morphological operations, and marked the region of the scar tissue on the endocardial wall of LV. Motivated by a Radon descriptor-based machine learning approach; the patches of 17 patients from Computer tomography (CT) images of the heart were used and categorized into “endocardial Scar tissue” and “normal tissue” groups. The ten feature vectors are extracted from patches using Radon descriptors and fed into a traditional machine learning model.

**Results:**

The decision tree has shown the best performance with 98.07% accuracy. This study is the first attempt to provide a Radon transform-based machine learning method to distinguish patterns between “endocardial Scar tissue” and “normal tissue” groups. Our proposed research method could be potentially used in advanced interventions.

## Introduction

Ventricular arrhythmia can lead to the formation of scar tissue in patients with myocardial infarction, which may have fatal consequences. Research from the World Health Organization (WHO) suggests that the number of individuals with cardiovascular disease (CVD) in the United States and other nations is projected to rise by 2.5 times by the close of 2050 [1, 2]. A rupture in the myocardium can occur during a myocardial infarction due to the formation of scar tissue on its wall. The function of the left ventricular (LV) pump is influenced by the size of the infarct. Consequently, the dimensions and coverage of the scar tissue play a pivotal role in determining the prognosis for patients who have suffered their first myocardial infarction. It’s essential to understand the LV remodeling process [3–7]. Excessive infarcts cannot endure the mechanical strains and are prone to rapid ruptures, leading to dire outcomes. Infrequent infarcts can result in a thinning of the myocardial wall, intensifying the stresses on the cardiac wall. Designing medications to enhance cardiac function or remodeling has proven to be a significant challenge.

This challenge arises due to the complexities associated with understanding the formation and properties of the infarct scar, as well as how its structure impacts cardiac mechanics, electrical conduction, and reflexive hemodynamic responses in clinical settings. Numerous innovative therapies are in development to evaluate scar tissue. In the computational domain, many studies have focused on detecting scars on the myocardium wall using magnetic resonance (MR) imaging rather than computer tomography (CT) modalities. In terms of algorithmic approaches, most have relied solely on the standard deviation and mean intensity value techniques for scar detection. Our preference for CT modalities stems from their speed and widespread availability. CT scans operate by capturing multiple X-ray images from different perspectives. We aim to bridge the gap between the Radon descriptor and machine learning algorithms in discerning patterns of normal and scarred tissue in the left ventricular endocardium. The subsequent sections of this document will detail our methods, provide a summary of our approach, and discuss the results and conclusions of our study.

## Methods

### Data acquisition and automatic segmentation of LV

The Philips computerized tomography device was used to capture cardiac CT images for 17 individuals (both males and females) with A-fib, aged between 50 and 62. This device recorded ten sets of timed frames at the same location using various contrast agents, spanning an entire cardiac cycle. Each data set comprised 409 images, each with a resolution of 512 × 512 pixels. We selected cardiac CT images with delayed enhancement for our dataset. After extracting the pertinent dataset, which included 149 CT images showcasing the LV portion, we omitted a few layers due to their irrelevance. The study adhered to the guidelines set by the Institutional Review Board of National Yang-Ming University Hospital, and all participants provided informed consent.

To automatically segment the LV, we employed the Segment CT software [5]. This software can pinpoint the LV on short-axis stacks by inserting reference points during the reconstruction process. We utilized mathematical calculations, specifically the standard deviation (SD) and the average pixel value, to identify potential scar areas in the dataset. This approach was informed by literature, which suggests that if the intensity value of a region exceeds three SDs above the mean intensity of a healthy myocardium, scar tissue is likely present [5, 8, 9]. Our focus was on assessing the pixel value of the LV myocardium wall region, as each pixel, represented in Hounsfield units (HU), offered valuable insights for our research. Literature indicates that HU provides a straightforward method for tissue characterization. The tissue density corresponds to the HU value, which in turn is directly related to the degree of x-ray attenuation for each pixel in the CT image [6, 10, 11]. In this phase, we automated the process of localizing and cropping the selected myocardium wall corresponding to the LV, and we calculated both the SD and average values.

### Implementation of morphological operations and patch creation

In this phase, we employed fundamental morphological operators such as erosion, dilation, opening, and closing to gather information [12, 15]. We established the criteria as follows: a pixel value that qualifies as a contrast area in the given dataset must exceed two standard deviations (SD) and one average value. With this approach, we were able to distinctly visualize the contrast areas. We then extracted 25 patches, each measuring 25 × 25 dimensions that encompassed both the scar region on the LV and areas of normal or non-scarred tissue.

### Radon descriptor and texture feature extraction

**Fig. 1:**
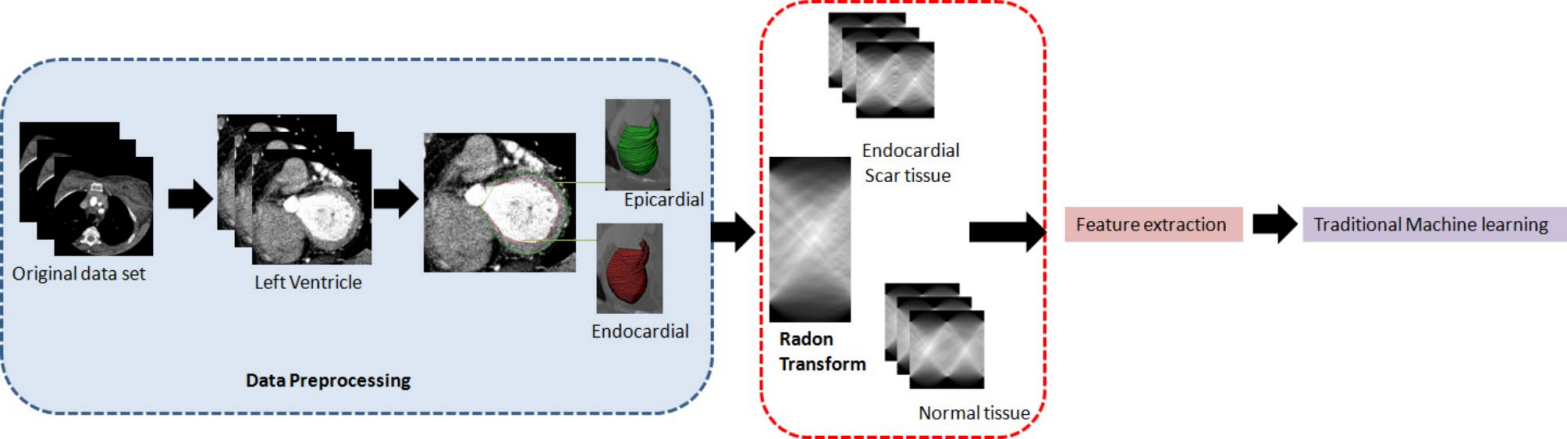
Workflow of predicting the left ventricular endocardial scar tissue pattern using Radon descriptor-based machine learning

The term “content-based image retrieval” (CBIR) denotes the process of searching for and analyzing the content within an image. This content can be identified using various criteria such as color, shape, texture, and more. CBIR is a pivotal aspect of computer vision research, especially in advanced medical systems that leverage human expertise to harness the computational power of computers [13]. The Radon transform, an integral transform, is applicable in CBIR endeavors. It determines the projection of an image in multiple directions [14]. This transform is adept at elucidating the visual characteristics of medical images [15, 16]. Challenges in this domain can be addressed using the Radon descriptor in conjunction with content-based medical image retrieval (CBMIR). After extraction, patches of each type were processed to generate Radon images for every patient using the Radon descriptor. For this purpose, we utilized the MATLAB R2018a platform in our study. By leveraging the local binary pattern (LBP) method for numerical data, we extracted texture features from the Radon images. This method is commonly employed in face detection and pattern recognition tasks. The LBP operator converts an image into a set of integer labels [15–18], which represent the image’s micro-features. These extracted numerical features were subsequently inputted into traditional machine learning models [Fig. 1].

## Results and discussion

In this study, we investigated an alternative method for automatically predicting endocardial Scar tissue pattern on the LV myocardial wall. According to published research, the function and remodeling of the LV endocardium are significantly influenced by the presence of scar tissue. Each slice’s average myocardium wall pixel value was computed, along with the SD, providing a total dataset average of 58.77 and an SD of 20.75. This was the most crucial step. We then calculated the average, SD, and number of pixels present in the specific growing area of the dataset, which is the myocardium wall of the LV, as well as the standard deviation for the entire population, and these values were found to be 59.89 and 21.2, respectively, using the region growing algorithm on the original dataset. This indicates that we conducted the analysis correctly because our results match those of the earlier studies [5, 8]. In the following phase, we ran the image processing morphological algorithm, which includes erosion, dilation, closing, and opening. We considered the threshold value of the intensity mean, which transforms the image into binary form. We discovered black and white spots, and white spots represent higher intensity which signifies a more high contrast area. Finally, we identified the location where there is a higher pixel value that would indicate the location of scar tissues. All endocardial Scar tissue and normal patches were then fed into the radon based machine learning to generate the Radon descriptors images. Numerical data of features were then extracted, and dissimilarity Wasserstein distance between normal and endocardial Scar tissue was calculated, giving a result of 9.05. These data were then sent to the supervised learning traditional models to fast prediction of the endocardial Scar tissue and normal/ non scar tissue pattern. The numerical feature data was fed into three classifiers: Decision Tree, Support vector machine (SVM), and Logistic Regression. A 5-fold cross-validation was used to evaluate the performances of these three algorithms. The development and performance of the model are carried out by the parameters like sensitivity, specificity, and accuracy.

Decision tree has shown the most promising results with 95.07% specificity, 96.08% sensitivity, and 98.07% accuracy with these hyper parameters (class_weight = classweight, max_depth = maxdepth, min_samples_split = minsamplessplit, min_samples_leaf = minsamplesleaf, max_leaf_nodes = maxleafnodes). The other classifier algorithms such as SVM and Logistic Regression have not shown good performance.

### Limitations of the study

Only a few A-fib patients were used in our study. The information was evaluated under the direction of cardiologists.

## Data Availability

The dataset of the current study is available from the first author on reasonable request.

## Abbreviations

LV: Left ventricular
CT: Computer Tomography
LBP: Local binary pattern
SD: Standard Deviation
HU: Hounsfield Units
